# Attitudes of Dutch general practitioners towards vaccinating the elderly: less is more?

**DOI:** 10.1186/s12875-015-0377-8

**Published:** 2015-10-28

**Authors:** Renske Eilers, Paul F. M. Krabbe, Hester E. de Melker

**Affiliations:** Department of Epidemiology, University of Groningen, Academic Medical Center Groningen, P.O. Box 30.001, 9700 RB Groningen, The Netherlands; Center for Infectious Disease Control, National Institute for Public Health and the Environment (RIVM), P.O Box 1, 3720 BA Bilthoven, The Netherlands

**Keywords:** General practitioners, Vaccine candidates, Interviews, Attitudes, Usefulness of vaccination

## Abstract

**Background:**

In many European countries, vaccinations are offered to the elderly. Expanding the programme to include routine vaccination against pneumococcal disease, herpes zoster, and pertussis, for example, could reduce disease burden amongst the growing population of persons aged 50 years and older. Since most countries involve general practitioners (GPs) in the programmes, the potential success of such new vaccinations depends on the attitude of GPs towards these vaccinations. This qualitative study explores Dutch GPs’ attitudes regarding vaccination in general, and their attitudes regarding the incorporation of additional vaccines in the current Dutch influenza vaccination programme.

**Methods:**

Interviews were held with ten Dutch GPs (five men and five women) that worked either in an academic hospital, in a practice based in a health center, or in individual practice. All interviews were recorded with a digital voice recorder and transcribed verbatim. Transcripts were analysed according to thematic analysis.

**Results:**

GPs perceived prevention as part as their job and believed vaccination to be effective for preventing infectious diseases. However, influenza vaccination was not always perceived as effective. Doubts regarding the usefulness of additional vaccinations were identified. If additional vaccines would be offered, this should be based on scientific evidence and the severity of the infectious disease. Selection of patients for vaccination should not be based solely on age, but more on risk factors. The GP should be the central point of contact for new vaccination campaigns; however, high workload was seen as a concern. Several GPs questioned their ability to refuse to distribute the vaccinations.

**Conclusions:**

A positive attitude towards implementing additional vaccinations is not apparent. Achieving the most health benefits seems to be the most important consideration of Dutch GPs regarding vaccinating older adults. Questions regarding the usefulness of vaccinating older adults should be taken into consideration. More research is necessary to confirm the results among a wider range of Dutch GPs.

## Background

Vaccinations are recommended for the elderly in many European countries. For example, in the Netherlands, a national influenza vaccination program is offered to persons aged 60 years and older [1]. In other countries, pneumococcal, diphtheria, tetanus and polio vaccination are also recommended [2]. There are reasons to expand these programmes, as demographic change will increase the elderly population size. In 2007, the Health Council of the Netherlands indicated that older age groups will be included in the target population for universal vaccination programs [3]. Additional routine vaccination against, for example, pneumococcal disease, herpes zoster, and pertussis could reduce the disease burden amongst those persons aged 50 years and older [4]. Several studies have indicated that herpes zoster and pneumococcal vaccines might be cost-effective for the older adult Western population [5, 6]. Herpes zoster vaccination is already recommended for people aged 70, 65, and 50 years and older in the United Kingdom, France, the Czech Republic and Austria, respectively. In Austria, pertussis vaccination is recommended for people aged 65 years and older [7].

In the Netherlands, the Dutch Ministry of Health, Welfare and Sport decides which risk groups are to be invited for the free influenza vaccination program, as advised by the Health Council of the Netherlands. The general practitioner’s office is the central point for organizing these immunization campaigns and the general practitioner (GP) (together with the practice nurse) selects, invites, and vaccinates the target population, accounting for 95 % of all vaccinations administered to risk groups. The remaining 5 % of vaccinations are given by others including a medical officer. Six percent of the target population had been recommended by a medical specialist instead of the GP; however, the vaccination itself was given by the GP [8].

The feasibility of extending current programmes thus depends on the willingness of the GPs to organize and endorse these campaigns. From literature on the attitudes of GPs and healthcare workers (HCWs), it is known that influenza and pneumococcal vaccinations for the elderly are considered important and are encouraged [9–12]. Certain concerns have also been reported: the possibility of negative side effects, low vaccine effectiveness, and perceived lack of consequences of the disease [13]. In addition, GPs identified their propensity to overlook vaccination due to their focus on acute medical problems [14]. They also noted several logistical and educational barriers, e.g. storage of and information about the vaccines [9].

Despite positive attitudes regarding the current vaccinations, little is known about GPs’ attitudes regarding vaccination of older persons in general, or regarding the potential candidate vaccines for this population. Given this lack of knowledge, the aim of this qualitative study was to explore these attitudes among Dutch GPs.

## Methods

### Selection of participants

Volunteer sampling was used to recruit the GPs. An information letter was sent to regional societies of general practitioners which was forwarded to affiliated practices. Unfortunately, the response rate was low. To include more GPs, practices across the Netherlands were selected randomly, taking into account location and type of practice, and approached directly by letter, followed by a phone call. If the attending GPs wished to participate, an appointment was made. In total, ten GPs agreed to be interviewed. All participants received a gift voucher following the interview. Informed consent was either obtained verbally by telephone or in writing by e-mail by the active enrolment of the GP’s for an interview. This type of study does not require ethics approval in the Netherlands because it does not fall under the Medical Research Involving Human Subjects act [15].

### Interview

The selected data collecting method was face-to-face interviews. Focus groups were not considered feasible due to the GPs’ high workload. Interviews were conducted in May and June 2013 at the general practitioner’s office, and lasted a maximum of 30 min (as informed beforehand). All interviews were conducted by the same researcher. Saturation was reached after eight interviews, meaning that after eight interviews, no new concepts emerged from the interviews. The conversation was based on a semi-structured topic list. This topic list was based on the literature and a focus group study among older adults in which the role of the GP was discussed. The open-ended questions covered four topics: 1) the perceived role of the GP concerning prevention in general; 2) his/her attitude regarding the current influenza vaccination programme; 3) his/her attitude towards herpes zoster, pneumococcal disease and pertussis, and reasons to vaccinate (or not) against these diseases; and, 4) the organization and practicality of vaccinating against additional infectious diseases. The potential candidates for immunization of persons 50 years and older were herpes zoster vaccine, pneumococcal vaccine and pertussis vaccine. Each interview started with discussion of vaccination from a broad perspective, and became more vaccination-specific towards the end. This was achieved by stating that besides influenza, herpes zoster, pneumococcal disease and pertussis were also prevalent in elderly persons. No more information was given. In general, influenza vaccination was discussed in the beginning of the interview, and later on the conversation focussed on the potential vaccine candidates. Following each interview, the GPs were given the opportunity to indicate if they felt any topics were missed regarding their attitudes towards the vaccination of older adults, and to contribute additional information. However, this was infrequently the case. All interviews were recorded with a digital voice recorder and transcribed verbatim.

### Analysis

The transcripts were analysed with the software program Nvivo (QSR International) and based on thematic survey principles [16]. Segments of text were coded by labeling passages with concepts abstracted from this text. Through an inductive process, main themes and related subthemes were identified based on these labelled passages. Themes were then redefined and reformulated to ensure that the different themes covered the context of the subthemes. In total, three rounds of coding were needed.

All interviews were conducted and initially coded by Renske Eilers (RE); one was coded by an independent researcher Irene Harmsen (IH) to minimize researcher bias. The results were compared, discussed and refined until consensus on the coding scheme and the labelling criteria was reached. The coding schemes of RE and IH were almost identical.

## Results

Ten GPs (five women and five men) of various ages (i.e. it was clear in the face-to-face interviews) were interviewed. All GP’s that were interested in the interview were interviewed. Unfortunately, no response rate can be provided because it was not exactly known how many GPs were notified by the regional GP societies. Three GPs worked in a general practice within an academic hospital; three in their own individual practice; and four in a practice based in a healthcare center. The mean interview time was 29 min. The nine themes derived from the interviews are presented below. The GPs’ attitudes regarding these themes are illustrated by quotes from the interviews.

### Prevention and the current influenza vaccination programme

This first section covers the attitudes on prevention in general and the current influenza vaccination program in the Netherlands.

#### Prevention in general

All ten interviewees agreed that prevention is part of a GP’s job, concurring that prevention forms an increasing part of their daily workload. This entails patient education and administration of preventive procedures (vaccination, measuring cholesterol). All GPs felt that vaccination was a good instrument to prevent infectious diseases. As one stated: *Once more, I believe it to be a very effective, inexpensive method to prevent lots of trouble and suffering. (GP 8. Male, Central region of the Netherlands, own practice)*.

#### Attitudes regarding the influenza vaccination programme

Even though GPs believed vaccination to be a good preventive tool, influenza vaccination was not always considered effective. Most considered the influenza vaccination programme useful: *I think that the influenza vaccination programme helps, that it would so to say help to prevent, well, a massive outbreak. (GP 6, male, Eastern region of the Netherlands, own practice)* Others did not: *Personally, I have the impression that the influenza vaccinations have no effect whatsoever, but of course that is not science, but that is not the impression of the people who get the shot, they manage to get through the winter better, so to say, they get through their year better. (GP 7, male, Central region of the Netherlands, own practice)* And even some who were positively inclined towards vaccination had reservations about lowering the age limit to 60 years, as was done in 2008 in the Netherlands: *But I do wonder about one thing, should it already be offered to people over 60, of course it used to be 65 (…)* continues*: We are all growing steadily older and we are, let’s say, relatively young for a longer time, so I find that a hard one to judge. (GP 4, female, Central region of the Netherlands, practice in healthcare center).*

### Potential vaccine candidates and the expansion of the influenza vaccination programme

#### Usefulness

In general, the idea of vaccinating older adults against additional infectious diseases was not well received. The majority of the GPs thought that availability in itself did not justify distributing the vaccine. Because they felt that experiencing disease is part of life, vaccination was not deemed a precondition for healthy ageing: *(…) yes, there is a tendency to try to avoid all of life’s risks and I can’t help but think that that is not how life is. (GP 1, female, Northern region of the Netherlands, practice in academic hospital).* They saw life as finite and death as a redeemer. As one GP said: *Once again, those who die of it* [the flu] *will be the weaker brothers and sisters, who are already confined to bed, or suffer from Parkinson’s or a serious case of COPD, or whatever. And then the end is actually merciful. (GP 7, male, Central region of the Netherlands, own practice)* Pneumococcal disease was also given as an example, calling it an old man’s best friend: *Oh, well no, I mean if you are 85 and your life isn’t rosy, or you have really had enough, pneumococcal disease, pneumonia, can be a kind way to depart. (GP 9, female, Central region of the Netherlands, practice in healthcare center).*

Expanding the range of vaccines in the programme could also have negative consequences on the perceived severity of a disease, as one GP mentioned: *Yes, and I also think, like from the moment that you offer vaccinations for shingles that -- oh, so shingles is apparently a serious illness. What I mean to say is that people’s perception will change. (GP 8, male, Central region of the Netherlands, own practice)*.

An epidemic is one situation for which adding vaccines to the programme could be justified. And some GPs saw the provision of additional vaccines as an advance in medicine, for obtaining health and healthy ageing.

### Considerations regarding potential vaccines

Two main arguments were identified for the way GPs expressed their willingness to consider potential new vaccines. These are ‘evidence-based practice’ and ‘severity of the infectious disease’.

#### Evidence-based practice

Evidence-based practice was considered very important, especially regarding a vaccine’s effectiveness and side-effects. Good vaccine characteristics formed a condition for implementing a new vaccine. As one GP explained: *You have to be sure it is useful, you have to be sure it helps, that it has no negative effects and that you can really prevent problems. (GP 5, male, Southern region of the Netherlands, practice in healthcare center)* Two others wondered whether vaccination could still benefit older adults, questioning how effective the immune response would be in an ageing immune system. If the vaccine could not prevent disease, it should at least reduce the disease burden. GPs discussed the balance between side-effects and vaccine effectiveness: *If there are a lot of mild or some serious side-effects, while the effectiveness is maybe not all that high, then I think, yeah, then you should not do this. The overall effect should be balanced. (GP 3, male, Northern region of the Netherlands, practice in academic hospital)* Although side-effects are no reason to advise against vaccination, they should be taken into account: *But I think that whatever you do, whatever interventions you make, you should also give due consideration to what the other side of it is, the side-effects, so the same goes for vaccinations.* (*GP1, female, Northern region of the Netherlands, practice in academic hospital)* If new vaccines were to be introduced, GPs assumed that implementation rested on thorough scientific evidence. As one GP stated: *Yes, certainly. But I just assume that that has been determined before it will even have been considered as an addition to the vaccination programme (…)* Continues: *The studies checking whether or not it is safe, I’ll take those for granted. (GP 4, female, Central region of the Netherlands, practice in healthcare center)* This assumption also applied to their role in informing patients: *Well, that is very important to me, I do want to be able to give a good, justified explanation to my patients that it is useful to do it, just like I would do with any medical interventions. (GP2, female Northern region of the Netherlands, practice in academic hospital)* Finally, to some GPs cost-effectiveness was an important aspect of evidence-based practice: *Right, and it should not be an afterthought that you have to consider the costs and benefits. We happen to live in times when we have to be frugal with every euro we spend. (GP5, male, Southern region of the Netherlands, practice in healthcare center).*

#### Severity of the infectious disease

The second important argument is the severity of disease. Although the initial disease burden of herpes zoster was perceived to be quite low, its consequences could be severe, justifying vaccination: *Essentially, the number of complaints that people have while they are suffering from that seems to be reasonable, as far as I can tell, but what counts is the number of complaints afterwards. (GP 3, male, Northern region of the Netherlands, practice in academic hospital)* Pneumococcal disease was considered severe enough to warrant vaccination due to its mortality rate.

Although pertussis was not perceived as severe enough among the elderly to justify vaccination, it was perceived as a threat to infants. In that light, ensuring herd immunity to protect children was considered a benefit of pertussis vaccination among the elderly. *Take pertussis, for instance, you do it above all to protect infants, to make sure that infants cannot get infected. (GP 5, male, Southern region of the Netherlands, practice in healthcare center)* The frequency of GPs encountering patients with a clinical syndrome during consultations appears to influence the GP’s perception of its severity. GPs were not often consulted for the three infectious diseases discussed here; herpes zoster was the one they saw most.

### Practical implications and implementation

GPs contemplated the practicalities of offering additional vaccines to older adults. In particular, they discussed the practicalities of adding new vaccines to the current influenza vaccination programme. They raised issues about the target population, the participating organizations, possible barriers, and autonomy.

#### Target population

The GPs felt that deciding who should receive the vaccination should not be based solely on age. Their reservations reflected the perceived lack of health benefits: *And again, that is the point, what do we gain by vaccinating the entire elderly population? (GP 2, female, Northern region of the Netherlands, practice in academic hospital)* It would be preferable to select people for vaccination on the basis of criteria such as co-morbidities to identify a high-risk population: *Of course, the benefit of the influenza vaccinations is the prevention of complications in vulnerable persons. And then if you start giving it to everyone -- well, if you and I get the flu, then it’s a nuisance, but you get over it. So there is no real need for a vaccination. (GP 5, male, Southern region of the Netherlands, practice in healthcare center).*

#### Participating organizations

All interviewees agreed that the GP’s office should be the central point for new vaccination campaigns. They dismissed the idea of leaving it up to other organizations such as the Public Health Service. As one GP stated: *Well that was clear then, at the time of that Mexican flu, that there was no organization, that is, apart from the GP’s office, that was able to carry out such a mass vaccination campaign in a very short time. So I think that should we do the preventive vaccinations, that the GP practice is the most suitable of all organizations, that is where it should be. (GP 5, male, Southern region of the Netherlands, practice in healthcare center).*

In addition, GPs felt that vaccinations should be given by a single organization: *I think it would be silly, going to your GP for your flu shot, but to the GGD* [Public Health Service] *for a vaccination for pneumococcal disease. (GP 10, female, Central region of the Netherlands, practice in healthcare center)* The latter respondent stated that even though the amount of work might triple by offering more vaccines, the GP should still be the preferred provider.

Two arguments were identified for this statement. First, using the GP’s practice ensures high coverage because GPs can effectively reach the target population. This is enhanced by the long-lasting patient-doctor relationship and the perceived prestige of GPs in the eyes of older adults that creates trust. The current influenza vaccination programme was taken as an example: *But well, I believe we can deliver that message -- like hey, it’s useful, just do it, yes -- better, I think, than anyone else in primary care, than the district health team. In general, we will have been in touch with the elderly for years, have treated them for years, so yes, alright, that implies we have built up trust, and that makes it rather easy to advise them, or means, for instance, that such advice will be taken. And that is what you see happen with the influenza vaccination. (GP 8, male, Central region of the Netherlands, own practice).* The second argument is more practical. If a vaccination programme is based on for example co-morbidity criteria (as preferred by the GPs), that selection process would necessarily involve the GPs: *It would not be very smart of the authorities if they would not involve the GP (…)* continues*; As far as I’m concerned, there is no other organization that knows the people as well as the GP. He is aware if the indications, has an exact registration of the medical history, and for the time being there is no other organization that can implement it. (GP 5, male, Southern region of the Netherlands, practice in healthcare center).*

Another role that the GPs identified for themselves is as the role of an advisor in their patients’ decision-making: *I’ll give advice, I won’t tell them what to do. No. (GP 4, female, Central region of the Netherlands, practice in healthcare center).* The role of the GP depends on the individual patient**:***Well, that is really just about what you do as a doctor and what the patient likes to see and get. And by now, I do know my elderly patients, and some, well they like to discuss things and then you go along, and others expect to get more directions, and then you tell them what they should do. (GP 3, male, Northern region of the Netherlands, practice in academic hospital).* Even if another organization were to be involved, GPs felt that they should have an important informative role to play because people would turn to them with their questions: *Well, I think that in that respect, seeing that all those people come to us to ask about it, that the patients think it is important what our opinion is. So in that respect, we have an important role in informing them (GP3 male, Northern region of the Netherlands, practice in academic hospital).*

#### Potential barriers

One potential barrier for adding additional vaccinations was the extra workload. Some GPs remarked that organizing the influenza vaccination programme alone is an extensive undertaking: *It does have a big impact, we have an influenza committee here, that starts up early on, all those people have to be selected. The GPs have to be chased to assess all those people. Altogether, it is a lot of bother. (GP 3, male, Northern region of the Netherlands, practice in academic hospital).* However, not all GPs felt that the extra workload would be insurmountable if financial compensation was forthcoming. As one GP stated: *Yes, barriers can be overcome in principle, but these tasks have to be facilitated, our professional group is naturally given quite a lot of tasks. (GP 1, female, Northern region of the Netherlands, practice in academic hospital).* Others, in contrast, believed that handling the distribution of the influenza vaccine would be easy, indicating that implementation of additional vaccines would not be a problem: *But really, that influenza programme, that runs very smoothly, it’s easy to carry it out, it is only one shot. So it takes two or three hours, really, per year, so it does not take much time. (GP 6, male, Eastern region of the Netherlands, own practice).*

Vaccine-specific selection criteria would add to the workload. To keep it within bounds, GPs would opt for similar selection criteria and combination vaccines. Therefore, they preferred any additional vaccines to be implemented within the influenza vaccination programme: *If the criteria would be the same that would make it a lot easier, and of course it would be best if they would both be simultaneous, like in both arms or as a cocktail vaccine like with hepatitis A and B. That would be handy, from a logistic point of view. (GP 3, male, Northern region of the Netherlands, practice in academic hospital)* Another GP put it succinctly: *I simply want to be able to order the right numbers on let’s say, the same website, that everything is just the same.* (*GP 4, female, Central region of the Netherlands, practice in healthcare center)* At the same time, the GPs foresaw restrictions on the patients’ freedom to choose a particular vaccine: *Well, see, assume that you could give it as one shot, then that would be easier, it would be less work, but then there might be people who say they want the one shot but not the other. I could imagine that that would complicate things. (GP 9, female, Central region of the Netherlands, practice in healthcare center).*

#### Autonomy

Several GPs questioned their ability to refuse to distribute the vaccinations, indicating that a positive attitude is not always necessary. They don’t believe that they have the authority to decide whether to vaccinate or not: *Well like that influenza vaccination, I am happy to leave that up to the authorities who in their wisdom have decided to adopt that programme -- oh well, then I’ll just go along with it, I’m just an employee, so to speak. (GP 7, male, Western region of the Netherlands, own practice)* Others did feel autonomous with respect to their own conditions: *If it is being offered, then it has been decided that it is worth it. Then I’ll just go along with it. But obviously, the costs should somehow be proportional to the benefits, the number of people that benefit from it, the disease burden. (GP 9, female, Central region of the Netherlands, practice in healthcare center).*

## Discussion

This qualitative study offers insight into the attitudes of Dutch GPs regarding the vaccination of older adults and regarding potential new vaccines candidates. Nine themes that reflect the attitudes of Dutch GPs were identified: prevention and the influenza vaccination programme; usefulness of additional vaccines; evidence-based practice; severity of the infectious disease; target population; participating organizations; potential barriers; and autonomy.

A positive attitude towards prevention in general and vaccination in particular does not necessarily imply a positive attitude regarding additional vaccinations. The interviews revealed several reasons why the GPs doubted the usefulness of potential vaccine candidates. First, they questioned eligibility for vaccination based solely on age criteria. Selection based on co-morbidity was considered more useful. Furthermore, they were concerned that implementation of a vaccine might cause the elderly to perceive certain infectious diseases as more serious. According to the interviewed GPs, the initial clinical syndromes of both herpes zoster and pertussis are not perceived as severe enough to warrant vaccination. Finally, they felt that it was not right to avoid all risks in life, not even the risk of death. In this light, pneumonia was seen as the old man’s best friend. The addition of other vaccinations to the influenza vaccination program may therefore involve ethical issues/challenges such as ageism (which is referred to as stereotyping or discriminating based on age), because implementation will most likely be age-dependent, and the ethics surrounding end-of-life decisions.

The influenza vaccination programme was initiated in 1997 and has since become common practice in the Netherlands [1]. Not all GPs had a positive attitude towards this vaccination programme. Nonetheless, distribution of the vaccine has become a routine part of their job. This ambivalence may emanate from their perception of themselves as not fully autonomous in the decision to give vaccinations. GPs comply with decisions made by the government, trusting that sufficient evidence has been gathered to justify the implementation of a new vaccine. Some GPs stated that certain conditions should be met, especially re-imbursement, to facilitate the distribution of the vaccines by the GPs.

Despite their ambivalent attitudes, they felt that if additional vaccines were introduced, GPs should be involved, regardless of their lack of a positive attitude or their high workload. Their role would be that of a consultant or advisor to the patients and/or as the organizer of the vaccination campaigns. In addition, they considered the GP office to be the most suitable place for distribution because it forms the locus of the patient-doctor trust relationship and the selection of patients eligible for vaccination.

Previous studies suggested that the GP plays an important role in the vaccination decision-making process of an older person [17]. GPs might play a key role in ensuring a high uptake of current and future vaccines. To this end, the support of the GPs should be optimized. This study suggests that their support could be won by providing them with appropriate information, given the importance they ascribe to evidence-based practice. Supply of information would also encourage a more positive attitude towards vaccination. That, in turn, would facilitate patient education and assist GPs in caring out their advisory role in the decision-making process of the elderly.

Qualitative studies on GPs’ attitudes towards (new) vaccines are scarce. Those that have been published focus on existing programmes and vaccination rates [18]. According to Swiss GPs, although pneumococcal disease was perceived as potentially severe, pneumococcal vaccination was found to be the least important vaccination in their daily practice, especially compared to influenza vaccination [18]. In our study, however, pneumococcal disease was identified as the most likely potential vaccine candidate when considering the attitude of the interviewed GPs [18]. Other studies have emphasized the importance of good vaccine characteristics [13, 19, 20], the disease burden caused by herpes zoster, specifically the longer-term consequences [20], the disease burden caused by pneumococcal disease [21], and the importance of vaccination as a preventive tool [22]. The literature on pneumococcal vaccination indicates that target population vaccination is preferred above general population vaccination based on a single age criterion [12, 13, 21, 23]. This conclusion is also drawn here.

Interestingly, the GPs interviewed in the present study questioned the usefulness of expanded immunization programmes for older adults. In the literature, this issue has only been addressed briefly by Van Haaren and not in other studies referenced within this article. The GPs interviewed in this study indicated that healthy elderly persons could withstand the flu, thus arguing against vaccinating the elderly against influenza [23].

### Study strengths and limitations

Given the low response rate, and consequently the low number of interviewed GPs, the present study was prone to selection bias. GPs have a high workload, and those GPs who were willing to participate might have had a strong interest in research or in elderly vaccination, or may have been critical of the topic. Also, GPs working an academic hospital were probably over-represented. Therefore questions might be raised concerning the representativeness of the study population. However, even though only 10 GPs were interviewed, they were located across the Netherlands, and saturation of the data was present after eight interviews. It is thus likely that the survey gathered all points of view. Nonetheless, the various attitudes identified in this study should be quantified.

## Conclusions

This qualitative study suggests that GPs do not necessarily have a positive attitude regarding the addition of potential vaccine candidates to the current Dutch influenza vaccination programme. The main challenge to expanding the vaccination programme to include potential vaccine candidates within the influenza vaccination schedule is how to achieve the most health benefits. Some questions about the usefulness of vaccinating remain unanswered. Thus, the degree of support among GPs, who are key players in the vaccination decision-making process of the elderly, is not yet clear. Further studies are needed to quantify the observations presented in this qualitative study, and to extend the research to encompass a larger sample of Dutch GPs.

## Abbreviations

GP: general practitioner
